# Short-Form Video Informed Consent Compared With Written Consent for Adolescents and Young Adults: Randomized Experiment

**DOI:** 10.2196/57747

**Published:** 2024-11-22

**Authors:** Aliyyat Afolabi, Elaine Cheung, Joanne Chen Lyu, Pamela M Ling

**Affiliations:** 1 Center for Tobacco Control Research and Education University of California San Francisco San Francisco, CA United States; 2 California Northstate University College of Medicine Elk Grove, CA United States

**Keywords:** health communication, video informed consent, randomized experiment, informed consent, adolescent, video, consent, e-cigarette, vaping, health research, social media, vaping cessation, smoking cessation

## Abstract

**Background:**

Adolescents and young adults have the highest prevalence of e-cigarette use (“vaping”), but they are difficult to enroll in health research studies. Previous studies have found that video consent can improve comprehension and make informed consent procedures more accessible, but the videos in previous studies are much longer than videos on contemporary social media platforms that are popular among young people.

**Objective:**

This study aimed to examine the effectiveness of a short-form (90-second) video consent compared with a standard written consent for a vaping cessation study for adolescents and young adults.

**Methods:**

We conducted a web-based experiment with 435 adolescents and young adults (aged 13-24 years) recruited by a web-based survey research provider. Each participant was randomly assigned to view either a short-form video consent or a written consent form describing a behavioral study of a social media–based vaping cessation program. Participants completed a postexposure survey measuring three outcomes: (1) comprehension of the consent information, (2) satisfaction with the consent process, and (3) willingness to participate in the described study. Independent sample 2-tailed *t* tests and chi-square tests were conducted to compare the outcomes between the 2 groups.

**Results:**

In total, 435 cases comprised the final analytic sample (video: n=215, 49.4%; written: n=220, 50.6%). There was no significant difference in characteristics between the 2 groups (all *P*>.05). Participants who watched the short-form video completed the consent review and postconsent survey process in less time (average 4.5 minutes) than those in the written consent group (5.1 minutes). A total of 83.2% (179/215) of the participants in the video consent condition reported satisfaction with the overall consent process compared with 76.3% (168/220) in the written consent condition (*P*=.047). There was no difference in the ability to complete consent unassisted and satisfaction with the amount of time between study conditions. There was no difference in the composite measure of overall comprehension, although in individual measures, participants who watched the short-form video consent performed better in 4 measures of comprehension about risk, privacy, and procedures, while participants who read the written document consent had better comprehension of 2 measures of study procedures. There was no difference between the groups in willingness to participate in the described study.

**Conclusions:**

Short-form informed consent videos had similar comprehension and satisfaction with the consent procedure among adolescents and young adults. Short-form informed consent videos may be a feasible and acceptable alternative to the standard written consent process, although video and written consent forms have different strengths with respect to comprehension. Because they match how young people consume media, short-form videos may be particularly well suited for adolescents and young adults participating in research.

## Introduction

Most standard informed consent processes use a written document that explains the purpose of the research, study procedures, the risks and benefits of the study, and alternative procedures and includes a contact person to answer questions about the research and participants’ rights. After reading the documents, participants sign or otherwise indicate that they understood the informed consent document. However, many consent documents are lengthy and contain complex terminology. Participants with less education or experience with research, including adolescents and young adults, may not understand, and they may skim or skip the consent document [1]. Even though the written informed consent procedure is valid, its length and use of terms unfamiliar to participants may result in misinterpretation of study procedures, or it may discourage participation. This, in turn, can lead to noncompliance, lower enrollment rates, and lack of generalizability of findings [1,2].

Systematic reviews of the literature have shown that using digital tools, such as video and audio platforms, for consent holds promise for improving comprehension of study information and potentially improving participation among people often underrepresented in research studies (eg, minors, young adults, participants from different cultural and religious backgrounds, non–English-speaking people, and people with disabilities) [3-5]. However, very few of these studies have been conducted with young people. One study comparing an iPad (Apple Inc) consent to a traditional written consent with parents and children found no difference for parents, but significantly greater understanding among the children in the iPad condition [6]. Another study of parents and adolescents found that a multimedia Microsoft PowerPoint presentation for permission or assent had significantly better comprehension than the paper process for both parents and children, with the most significant differences for adolescents [7]. In addition, a study of informed consent for a clinical study of lung disease found that participants who viewed interactive videos had equivalent comprehension and greater satisfaction compared with those who read the standard informed consent document [8,9].

Media has changed substantially since these studies were published, and social media videos are increasingly popular among adolescents: in 2022, 95% of adolescents reported watching YouTube (Google) videos, and 67% used TikTok (ByteDance) [10]. Although multimedia video consent has been shown to serve as an effective alternative to standard written consent [11], the length and format of the multimedia consent in previous studies are different from those on social media platforms popular among adolescents and young adults. Social media videos are short: TikTok videos’ average length between August 2022 and January 2023 was 32-42 seconds depending on account size [12]. One study that investigated student engagement with educational videos found that shorter educational videos between 0 and 3 minutes had the highest engagement rate compared with educational videos longer than 6 minutes [13]. This study also found that students engaged more when the speaker addressed the camera with intentional eye contact compared with other videos, such as Microsoft PowerPoint slides and digital tablet drawings with voiceovers. Another study that examined the social media short-form videos’ effect on youth well-being found that younger people under the age of 22 years spent more time watching short-form videos and were more satisfied with entertainment and relaxation-themed short-form videos [14]. Despite the popularity of short-form videos among adolescents and young adults, they have not been studied as a format for informed consent in this young priority population. This study fills that gap. We tested the effectiveness of a short-form video consent compared with the standard written consent among adolescents and young adults in terms of comprehension, satisfaction, and willingness to participate in a hypothetical study of a vaping cessation program.

## Methods

### Study Procedure and Participants

**Figure 1:**
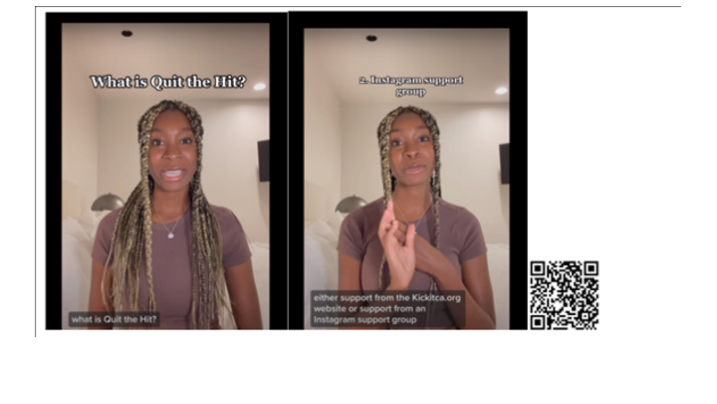
Short-form video consent screenshots showing eye contact, gestures, and facial expression. QR code links to the short-form video consent.

We performed a randomized experiment through a 1-time, web-based survey with 435 participants between the ages of 13 and 24 years. Participants were recruited by a commercial research company, Generation Lab, using standard recruitment procedures such as member referrals, email lists, and social media ads. The inclusion criteria for our study were (1) English literacy, (2) aged 13-24 years, and (3) having access to a computer or mobile phone with the capacity to play videos and complete the web-based survey. After signing consent for the study, participants were randomly assigned to 1 of 2 groups: the short-form video consent group (experimental condition) or the written consent group (control condition). Participants in the experiment group watched a 90-second consent video that contained information about a hypothetical behavioral study about a vaping cessation program; The video content had a young adult English speaker directly addressing the video consent viewer with clear message delivery and variable tones of voice (Figure 1). The formatting style was similar to TikTok social media videos. The control group read a written document that contained the same information about the behavioral study. Immediately after exposure to the informed consent, participants completed a questionnaire that measured participants’ comprehension, satisfaction, and willingness to participate in the described study.

### Ethical Considerations

The study was approved by the University of California San Francisco institutional review board (Protocol #23-38556). All participants completed a written (delivered via a web-based survey platform) informed consent for participation. Generation Lab managed contact with study participants, and only deidentified data files were shared with the study team for analysis. Participants were compensated US $5 to US $8 through gift cards administered by Generation Lab. No research participants were identifiable in the paper; the video screen grabs in Figure 1 contain images of the lead author (AA), and she has given permission for the use of these images.

### Outcome Measures

The postexposure survey included multiple-choice questions addressing 3 outcome variables: comprehension of the consent information, satisfaction with the consent process, and willingness to participate in the described study.

#### Comprehension of the Consent Information

Comprehension measures consisted of 5 main questions adapted from previous research [8], including 11 individual items measuring overall study purpose and length, for example, “What is the study about?” (multiple choice) and “If I choose to participate, I am required to remain in the study for the full 6 months” (true or false); 3 items addressing study risks; and 5 items addressing specific study procedures. For each of the 11 items, a correct answer was coded with “1” and an incorrect answer was coded with “0.” For “select all that apply” questions with multiple correct answers, we coded each option as a separate item with “1” for a correct response and “0” for an incorrect response. The overall comprehension score was calculated by adding up the number of correct answers (range 0-11).

#### Satisfaction With the Consent Process

This was assessed by 3 questions adopted from a previous study [8]: “How satisfied were you with your ability to complete the consent process for this research study on your own without any staff?” “How satisfied were you with the time required to complete the consent process?” and “How satisfied were you with the overall consent process?” Response options for these 3 questions were on a 7-point Likert scale, consistent with the previous study using this measure [8]. Answers were coded with a value of 1 representing completely dissatisfied and a value of 7 representing completely satisfied, with a score of 5 or greater indicating satisfaction.

#### Willingness to Participate in the Described Study

This was assessed by a single question: “Based on the consent you viewed, if you qualified for the study, how likely would you participate in this study?” with 5 Likert response options: “1=Very unlikely,” “2=Unlikely,” “3=Neither likely nor unlikely,” “4=Likely,” or “5=Very likely.”

Sociodemographic characteristics included age, gender, sexual orientation, race, education level, and health literacy. Health literacy was assessed by the question: “How often do you need to have someone help you when you read instructions, pamphlets, or other written material from your doctor or pharmacy?” [15]. Response options for the health literacy question were coded “5=Never,” “4=Rarely,” “3=Sometimes,” “2=Often,” “1=Always,” with a value of 4 or 5 representing the high level of health literacy and <4 representing the low level, consistent with prior research [15]. Vaping history was measured with an optional question: “Have you ever vaped nicotine in your life?” with response options “yes” and “no.”

### Statistical Analysis

All responses were coded, and SPSS Statistics (IBM Corp; version 26) was used for data cleaning and analysis. After receiving 435 survey responses, the dataset was screened by Generation Lab for missing data and outliers. Study completion time was calculated after extreme outliers were removed using the standard 3IQR rule, where any value greater than 3IQR greater than the third quartile or less than 3IQR less than the first quartile is designated as an extreme outlier [16]. Following these criteria, Generation Lab removed 24 outlier cases in the written consent group and 31 outlier cases in the short form consent video group from the study sample to calculate the average study completion time; no cases were removed due to missing data or unanswered question items, and no cases were excluded from analysis. Descriptive statistics were computed for the demographics of the overall sample, written consent group, and short-formed consent video group. A series of independent samples 2-tailed *t* tests and chi-square tests were used to assess the homogeneity of participant characteristics between the 2 groups and compare outcomes (comprehension, satisfaction, and willingness to participate) between the 2 groups. Because participants of different ages or with experience with vaping might have more familiarity with the topic or more willingness to participate in the study, we conducted prespecified subgroup analyses to examine if there were differences in the outcomes between adolescents and young adults, as well as among those with vaping experience. The level of significance for all analyses was set at *P*<.05.

## Results

### Participant Characteristics

Of the 435 participants, 215 (49.4%) were randomized to the video consent group and 220 (50.6%) were randomized to the written consent group. There was no statistical difference in characteristics between the 2 groups (all *P*>.05; Table 1). The average age of the participants was 18.67 (SD 2.69) years. For gender identity, 49% (213/435) of the participants identified as male; 40.9% (178/435) as female; and 10% (44/435) as nonbinary, transgender, queer or other identity. For race or ethnicity, 41.4% (180/435) identified as non-Hispanic White; 24.6% (107/435) identified as non-Hispanic Black; 13.1% (57/435) identified as non-Hispanic Asian; 14.3% (62/435) identified as Hispanic; and 5.3% (23/435) identified as other or multiracial. For education, participants ranged from 9th grade through college, with the largest group (94/435, 21.6%) being in 12th grade. The majority of the participants (369/435, 84.8%) self-reported a high level of health literacy. In addition, 49.4% (215/435) of the participants reported experience with vaping at some time in their life (Table 1). Excluding extreme outliers, the average survey completion time was 4.5 (SD 2.49) minutes for the video consent group and 5.1 (SD 3.4) minutes for the written consent group.

**Table 1:** Demographic characteristics, health literacy, vaping experience, and study completion time by condition.

Characteristics (demographics)			Total (N=435)		Video consent group (n=215)		Written consent group (n=220)		Chi-square or 2-tailed *t* test (*df*)			*P* value	
**Age, mean (SD)**			18.67 (2.69)		18.54 (2.65)		18.79 (2.73)		0.97 (433)^a^			.37	
**Gender, n (%)**										7.16 (6)^b^			.52
	Men	213 (49)		104 (48.4)		109 (49.5)							
	Women	178 (40.9)		84 (39.1)		94 (42.7)							
	Nonbinary	18 (4.1)		12 (5.6)		6 (2.7)							
	Trans woman	3 (0.7)		2 (0.9)		1 (0.5)							
	Trans man	10 (2.3)		5 (2.3)		5 (2.3)							
	Gender fluid or queer	5 (1.1)		3 (1.4)		2 (0.9)							
	Other	8 (1.8)		5 (2.3)		3 (1.4)							
**Race or ethnicity, n (%)**										15.31(5)^b^			.36
	Non-Hispanic White	180 (41.4)		99 (46.0)		81 (36.8)							
	Non-Hispanic Black	105 (24.1)		49 (22.8)		56 (25.4)							
	Non-Hispanic Asian	59 (13.6)		25 (11.6)		34 (15.5)							
	Hispanic	62 (14.3)		32 (14.9)		30 (13.6)							
	Other or multiracial	23 (5.3)		7 (3.3)		16 (7.3)							
	Prefer not to answer	6 (1.4)		4 (1.9)		2 (0.9)							
**Education, n (%)**										5.96 (7)^b^			.82
	6th grade	1 (0.2)		0		1 (0.5)							
	9th grade	14 (3.2)		4 (1.9)		10 (4.5)							
	10th grade	35 (8.0)		18 (8.4)		17 (7.7)							
	11th grade	56 (12.9)		28 (13.0)		28 (12.7)							
	12th grade	94 (21.6)		51 (23.7)		43 (19.5)							
	Graduated high school or GED^c^	55 (12.6)		30 (14.0)		25 (11.4)							
	College	152 (34.9)		71 (33.0)		81 (36.8)							
	Other	28 (6.4)		13 (6.0)		15 (6.8)							
**Health literacy, n (%)**										0.40 (1)^b^			.69
	Low	66 (15.2)		34 (15.8)		32 (14.5)							
	High	369 (84.8)		181 (84.2)		188 (85.5)							
**Lifetime nicotine vaping experience^d^** **(yes), n (%)**			215 (49.4)		103 (47.9)		112 (50.9)		0.73 (2)^b^			.69	
**Average completion time (minutes)^e^** **, mean (SD)**			4.8 (3.0)		4.5 (2.49)		5.1 (3.40)		2.1(378)^a^			.04	

^a^2-tailed *t* test.

^b^Chi-square test.

^c^GED: General Education Development.

^d^Lifetime nicotine vaping experience was calculated based on the 309 participants who answered this optional question.

^e^Average study completion time was calculated from 380 participants, eliminating 55 extreme outliers—24 in the written group and 31 in the video group.

### Informed Consent Comprehension

There was no significant difference in the overall comprehension score (range 0-11) between participants in the video consent group and those in the written consent group (8.84 vs 8.62; *P*=.95). However, participants in the video consent group performed better than those in the written consent group on 4 individual measures of comprehension, including 1 item on the topic of the study, 2 potential risks, and 1 study procedure (Table 2). On the other hand, for 2 items in the study procedure, the percentage of participants who correctly answered was significantly higher in the written consent group (*P*=.02 and *P*=.03; Table 2).

**Table 2:** Percentage of participants in the short-form video consent group and written consent group that answered the comprehension questions correctly.

Question		Correct answers, n (%)		Chi-square (*df*)	*P* value
		Video consent group (n=215)	Written consent group (n=220)		
**1. What is the study about? (vaping of nicotine and/or cannabis)**		215 (100)	216 (98.2)	3.95 (1)	.047
**2. True or False? If I choose to participate, I am required to remain in the study for the full 6 months (false)**		184 (85.6)	186 (84.5)	0.09 (1)	.76
**3. True or False? If I choose to participate, I must complete all of the study surveys (false)**		172 (80)	178 (80.9)	0.06 (1)	.81
**What are some of the possible risks associated with participating in this study? (Select all that apply)**					
	4. There are no risks associated with participating in this study (false)	175 (81.4)	163 (74.1)	3.35 (1)	.07
	5. There is a small chance that my participation in this study can cause a loss of privacy (true)	127 (59.1)	109 (49.5)	3.97 (1)	.046
	6. Some of the questions may make me feel uncomfortable (true)	153 (71.2)	137 (62.3)	3.87 (1)	.049
**What will happen if you participate in the study? (Select all that apply)**					
	7. I will join a group to quit vaping on Instagram (true)	193 (89.8)	175 (79.5)	8.72 (1)	.003
	8. I will be randomly assigned to an Instagram group or a website to quit vaping (true)	113 (52.6)	128 (58.2)	1.39 (1)	.24
	9. I will complete one survey every day during the study (false)	181 (84.2)	201 (91.4)	5.24 (1)	.02
	10. I will complete surveys now and at 1, 4, and 7 months (true)	179 (83.3)	199 (90.5)	4.95 (1)	.03
	11. I will be paid only if I quit vaping (false)	208 (96.7)	209 (95)	0.83 (1)	.36

### Prespecified Subgroup Analyses

We further conducted subgroup analyses. Overall comprehension was checked within adolescents (aged 13-17 years) and young adults (aged 18-24 years), and there were no significant differences in overall comprehension by consent group among adolescents (*P*=.17) or young adults (*P*=.59). We also compared comprehension levels among participants who reported lifetime experience with vaping (n=215). Among participants with vaping experience, those in the video group had a significantly higher percentage of correct answers for 3 items: “there are no risks associated with participating in this study” (*P*=.004), “there is a small chance that my participation in this study can cause a loss of privacy”(*P*=.046), and “some of the questions may make me feel uncomfortable” (*P*=.01), compared with participants in the written consent group with vaping experience.

Participants in both the video consent group and the written consent group reported high levels of satisfaction (Table 3). Combining the 3 measures of satisfaction, there was no significant difference in the overall satisfaction between the video consent group and written consent group (5.97 vs 5.83; *P*=.65). However, on the single item, “How satisfied were you with the overall consent process?” the video consent group scored significantly higher than the written consent group (5.80 vs 5.47; *P=*.01). A total of 83.2% (179/215) of respondents in the video condition and 76.3% (168/220) in the written consent condition reported satisfaction with the overall consent process. There was no significant difference in willingness to participate between the short-form video consent group and written consent group (4.21 vs 4.24; *P*=.82). Both groups reported high willingness to participate in the hypothetical study described in the consent forms, with a score of 4.21 for the video consent group and 4.24 for the written consent group, indicating that they were “likely” or “very likely” to participate if they qualified for the study.

**Table 3:** Satisfaction score in the study by condition.

Satisfaction	Video consent group, mean (SD)	Written consent group, mean (SD)	2-tailed *t* test (*df*)	*P* value
1. How satisfied were you with your ability to complete the consent process for this research study on your own without any staff? (scale 1 to 7)	6.09 (1.23)	6.14 (1.04)	0.40 (433)	.69
2. How satisfied were you with the time required to complete the consent process? (scale 1 to 7)	6.01 (1.33)	5.88 (1.28)	–1.02 (433)	.31
3. How satisfied were you with the overall consent process? (1 to 7)	5.80 (1.61)	5.47 (1.81)	–1.99 (433)	.047
Overall satisfaction (1 to 7)	5.97 (1.13)	5.83 (1.10)	–1.29 (433)	.20

## Discussion

### Principal Findings

This study is among the first to explore the effectiveness of using short-form consent videos that mimic popular social media videos compared with a standard written consent among adolescents and young adults. Our findings suggest that short form informed consent video delivered information as well as standard written consent forms and performed significantly better on some individual measures of comprehension and satisfaction. In addition, the short-form video consent group had a slightly shorter average completion time compared with the written consent group. Participants who watched the short-form informed consent video had similar comprehension compared with the participants who read the written consent document. Participants in the short-formed video consent group answered an average of 80.3% (SD 0.18%) of the 11 comprehension questions correctly, indicating a reasonable understanding of the consent information, similar to participants who read written consent documents (78.6%, SD 0.18%). This finding was consistent with previous multimedia studies. One clinical study of lung disease found that the video consent format led to equivalent comprehension and greater satisfaction among participants who saw a virtual multimedia informed consent compared with the participants who read the standard informed consent document [8,9]. Another clinical study examining the use of video consent in adolescents and adults in prison found that adult participants in the audiovisual groups had a significantly better understanding than those who read the paper consent document. In the adolescent group, there was no significant difference in the understanding and evaluation of the consent information [17]. Although these findings were consistent with our results, our study design and population differed. Both previous studies used videos that were 4-minute animated slide shows with voiceovers. In addition, our study focused only on adolescents and young adults aged 13-24 years.

While the shorter video showed equivalent comprehension to the written consent document, the short format is relatable and similar to what adolescents and young adults may regularly encounter on popular social media sites [10]. It is worth noting that the short-form video in this study matched the topic of the hypothetical study (a social media vaping intervention study) and the study procedures were low risk and straightforward. Previous studies of video consent were for complex procedures, such as in vitro fertilization or Mohs surgery, or high-risk situations, such as care in the intensive care unit, where longer and more detailed videos are likely necessary [6,9,18]. Our findings for short-form video are most likely to apply to low-risk behavioral studies among younger healthy volunteers.

While the overall measure of comprehension was not different between the 2 conditions, on 4 of the individual items, participants in the short-form video consent group performed better than participants in the written consent group. Participants in the short-form video consent group had a better understanding of what the study was about generally, two risks associated with the study, and 1 measure of study procedures. On the other hand, participants in the written consent group had a better understanding of 2 measures of the study procedures.This differs from previous studies that found that those who underwent multimedia informed consent had a better understanding of the use of their personal health information and how to withdraw from the study, compared with their counterparts who underwent traditional paper consent [19-21]. Our findings suggest that different formats may communicate different aspects of study information more effectively, and perhaps both video and written consent forms should be available to study participants.

In our study, on 1 question addressing randomization, “What will happen if you participate in the study? (Select all that apply),” we found that participants in both groups had difficulties selecting the correct response—“I will be randomly assigned to an Instagram group or a website to quit vaping.” Approximately half of the participants in both groups selected the incorrect response. This finding suggests that some survey questions may be difficult for participants to understand regardless of the format of the consent process. This difficulty may also have been due to the phrasing of the question. Participants were asked to “select all that apply” from a list of statements, and there were 2 similar answer choices for this question: “I will join a group to quit vaping on Instagram” and “I will be randomly assigned to an Instagram group or website to quit vaping.” The similarity of the wording of these 2 items might have led to fewer participants answering correctly (by selecting both from the list) than if each item had been asked as a separate true-or-false question. Pilot-testing can serve as a valuable tool for identifying challenging questions and for exploring effective information delivery formats to ensure clear communication with participants.

The topic of our hypothetical study was a social media intervention for adolescents and young adults who may be interested in quitting vaping. Our subgroup analysis among participants with vaping experience found no overall difference in comprehension, although those who viewed the video consent had significantly better comprehension on 3 measures compared with those who read the written consent form. This suggests that short-form video would be acceptable for young people likely to qualify for the study.

Participants who viewed the short-form informed consent video were more satisfied with the overall consent process. This may be because the consent video was more relatable and engaging. The speaker in the video was young and similar to the age of the participants and used different vocal tone modulations to capture attention. In addition, the video was less than 2 minutes long and had a 9:16 format, compatible with mobile phones. The higher satisfaction is consistent with other studies that found that multimedia delivery consent forms led higher satisfaction in participants [6,19,22]. Although self-reported satisfaction with the time required to consent was not significantly different, participants who viewed the short-form informed consent video had a faster average survey completion time. Higher overall satisfaction might be related to either increased time efficiency or a subjective sense of ease in the process. In addition, based on the learning and communication literature, several additional factors could serve as potential moderators for study comprehension or satisfaction and could be addressed in future research. For example, according to the media-richness theory, face-to-face communication is the richest medium because the use of body language, facial expressions, and tone of voice mirrors the natural language and conveys emotion [23]. How these features of short-form videos impact comprehension or satisfaction may warrant future research. Other studies of multimedia learning have suggested that both gender and spatial abilities can influence learning outcomes, and these could be addressed in future research [24]. In addition, studies could address visual signals such as facial expression, body postures, and vocal tones that may influence the perceived credibility and trustworthiness of the presenter, and other nonverbal signals that communicate dominance, composure, or trust that could enhance participants’ understanding and willingness to participate in research [25,26]. Future research studies could explore more of these specific factors and their influence on satisfaction and understanding in the context of informed consent.

### Limitations

Our study has limitations. First, the respondents were aware they were taking part in a research study to rate different delivery methods of consent information, which may have led to social desirability bias. Second, our study’s survey environment differed from the real-world environment in which people read an informed consent without comprehension tests. Participants may have paid increased attention to the consent content in order to correctly answer comprehension questions, which may be less likely to happen in real situations. Third, 84.8% (369/435) of the participants in our research study self-reported high health literacy; thus, the findings may not generalize to those with low health literacy. Video consent has the potential to improve comprehension for individuals with low literacy, and future studies should be conducted with participants with low literacy. Finally, this study focused on short-form video consent for a low-risk behavioral study in English-speaking young adults and adolescents. Future research could test the shorter-form video consent with different types of studies and with older or non–English-speaking people.

### Conclusion

Short-form video consent for low-risk behavioral studies had similar comprehension and satisfaction compared with standard written informed consent among adolescents and young adults, so it is an acceptable alternative to written forms. Because they match the format of information widely used by young people, short-form video consent may make the research enrollment process more acceptable for this priority population. This study contributes to a growing body of evidence in support of consent videos. Researchers should continue to explore creative consent formats that match their participants’ needs.

## Data Availability

The datasets generated during and/or analyzed during this study are available in the Dryad repository [27].
